# Vitamin D receptor gene polymorphisms and risk of intervertebral disc degeneration: An updated meta-analysis based on 23 studies

**DOI:** 10.1097/MD.0000000000025922

**Published:** 2021-05-21

**Authors:** Jing Xue, Yueming Song, Hao Liu, Limin Liu, Tao Li, Quan Gong

**Affiliations:** From the Department of Orthopaedics, West China Hospital, Sichuan University, Chengdu, China.

**Keywords:** disc degeneration, meta-analysis, polymorphisms, systematic review, vitamin D receptor

## Abstract

**Background:**

Numerous studies have investigated the associations between Vitamin D receptor (VDR) gene polymorphisms and risk of intervertebral disc degeneration but the results remain controversial. This study aimed to drive a more precise estimation of association between VDR gene polymorphisms and risk of intervertebral disc degeneration.

**Methods:**

PubMed, EMBASE, Cochrane library, Web of Science and China Knowledge Resource Integrated Database for papers on VDR gene polymorphisms and risk of intervertebral disc degeneration were searched. The pooled odds ratios (ORs) with 95% confidence intervals (CIs) were used to assess the strength of association in the homozygote model, heterozygote model, dominant model, recessive model and an additive model.

**Results:**

Overall, 23 articles were included in the final meta-analysis. The subgroup analyses by ethnicity showed a significant association of VDR FokI mutation with disc degeneration risk in Caucasians (recessive model, OR with 95%CI 1.301, [1.041, 1.626]; additive model, OR with 95%CI 1.119, [1.006, 1.245]). The results of subgroup analyses by ethnicity showed a significant association of VDR TaqI mutation with disc degeneration risk in Asians but not in Caucasians. There was a significant association between VDR ApaI mutation and risk of disc degeneration and subgroup analyses by ethnicity showed a significant association in Caucasians and in Asians.

**Conclusions:**

In summary, VDR FokI polymorphisms was associated with disc degeneration risk among Caucasians but not Asians, VDR TaqI polymorphisms was associated with disc degeneration risk among Asians but not Caucasians, while VDR ApaI polymorphism was associated with disc degeneration risk among Asians and Caucasians.

## 1 Introduction

Low-back pain is a common musculoskeletal problem leading to work disability and heavy healthcare costs at present.^[1]^ It was reported that 50–80% of adults may suffer from at least one episode of back pain during their lifetime.^[2]^ As a major cause of back pain, the mechanism of disc degeneration has not been fully understood and has been commonly accepted as a “multi-factorial” result, where lifestyle, individual genetic background and environmental risk factors are involved.^[3]^ However, the exact etiology of disc degeneration remains unknown and recent studies supported that genetic factors may play a crucial role in the occurrence and development of disc degeneration.^[4]^

Vitamin D receptor (VDR) gene is one of the most studied candidate genes associated with disc degeneration, which is located on chromosome 12q12–q14 with eight protein-coding and six untranslated exons.^[5]^ Allelic variants of the gene encoding VDR, include TaqI (rs731236), FokI (rs2228570) and ApaI (rs7975232) have been reported to be associated with disc degeneration but still remains controversial. As the previous studies have generally been small-sized, several meta-analysis have been performed to explore the association between VDR gene polymorphisms and disc degeneration risk. Xu et al.^[6]^ performed a meta-analysis and reported that the VDR (TaqI, FokI, ApaI) gene polymorphisms were not significantly associated with the risk of disc degeneration. Zhao et al.^[7]^ performed a meta-analysis and found that FokI polymorphism is not generally associated with disc degeneration, but there is increased risk for disc degeneration in Hispanics and Asians carrying FokI allele T. Several meta-analyses were performed subsequently but the conclusions still remains controversial.^[8–12]^ After that a series of novel studies have been performed, so an updated meta-analysis based on 23 studies was performed to clarify the effect of VDR gene polymorphisms (TaqI, FokI and ApaI) on the risk of disc degeneration.

## 2 Materials and methods

### 2.1 Search strategy

For Systematic Reviews and Meta-Analyses, the study does not require approval by the ethics committee. This meta-analysis was performed according to the standard MOOSE guideline.^[13]^ PubMed, EMBASE, Cochrane library, Web of science and China Knowledge Resource Integrated Database (until April 1, 2020) were searched using search terms as “(“Vitamin D receptor” OR VDR OR TaqI OR FokI OR ApaI OR rs731236 OR rs2228570 OR rs7975232) AND (polymorphism OR variants OR mutation) AND (“disc degeneration” OR “low back pain”).” Studies published in English or in Chinese language were selected. Case–control studies containing available genotype frequencies of TaqI, FokI and ApaI were chosen. Related reference articles were also searched to identify other relevant publications. The study with largest sample size was selected if more than one article were published using the same case series. Unpublished data were not included.

### 2.2 Inclusion and exclusion criteria

Eligible studies were selected following inclusion criteria:

(1) VDR gene (TaqI, FokI and ApaI) polymorphisms and disc degeneration;
(2) human case-control design;
(3) studies that reported the frequency of TaqI, FokI and ApaI polymorphisms; and
(4) published in English or Chinese.

**Figure 1:**
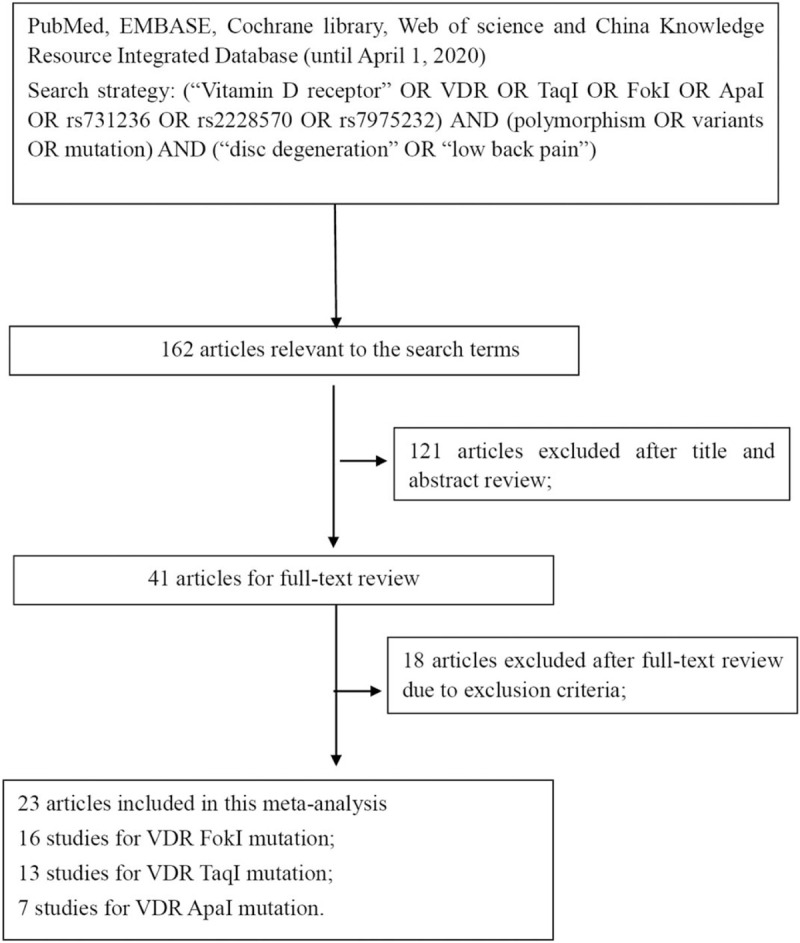
Study identification flowchart.

The criteria for the exclusion of studies are as follows:

(1) not a primary case-control study;
(2) no usable or sufficient genotype data reported;
(3) studies whose allele frequency in the control population deviated from the Hardy–Weinberg Equilibrium (HWE) at a p value equal or less than 0.01;
(4) case reports, letter to Editor, book chapters or reviews. The study inclusion and exclusion procedures are summarized in Fig. 1.

### 2.3 Data extraction

Two investigators independently extracted the data from all included studies according to the inclusion and exclusion criteria listed above. Discrepancies were solved through discussion with another investigator. The following information was extracted: the first author's name, year of publication, the country in which the study was conducted, the source of control group evidence of HWE in controls, the sample size, allele/genotype frequencies.

### 2.4 Statistical analysis

STATA software Version 15.0 (Stata Corp LP) was used for all statistical analyses and P values less than 0.05 were considered statistically significant. Odds ratios (ORs) with 95% confidence intervals (CIs) were used to assess the strength of association between VDR gene polymorphisms and disc degeneration risk. The HWE tests were performed on control groups using a Pearson's goodness-of-fit chi-square. The pooled OR was calculated by a fixed-effect model or a random-effect model according to the heterogeneity. The pooled ORs were calculated for the homozygote model, heterozygote model, dominant model, recessive model, and an additive model. Cochran *Q*-statistic and the *I*^2^ metric were conducted to assess heterogeneity between studies, *P* < .10 and *I*^2^ > 50% were considered statistically significant.^[14]^ Sensitivity analyses were also performed after sequential removal of each study. Lastly, Begg's funnel plot and Egger test were used to examine statistically any publication bias.

## 3 Results

### 3.1 Characteristics of the included studies

According to the inclusion and exclusion standard, a total of 23 studies^[15–37]^ published from 2003 to 2019 were included in this meta-analysis: 16 studies with 2109 cases and 2454 controls for VDR FokI mutation and risk of disc degeneration; 13 studies with 1918 cases and 2019 controls for VDR TaqI mutation and risk of disc degeneration; 7 studies with 1152 cases and 1251 controls for ApaI mutation and risk of disc degeneration. The genotype distributions in the controls for all studies were consistent with the Hardy-Weinberg equilibrium. The characteristics of all included studies are summarized in Table 1.

**Table 1:** The characteristics of all included studies.

				Case				Control				
Study	Year	Region	Ethnicity	Total	11	12	22	Total	11	12	22	HWE
FokI												
Yang et al.	2019	China	Asian	454	122	207	125	485	126	225	134	0.113
Ozdogan S, et al.	2019	Turkey	Caucasian	45	3	11	31	49	6	22	21	0.949
Mashayekhi et al.	2018	Iran	Caucasian	180	64	86	30	230	106	104	20	0.436
Withanage et al.	2018	Sri Lanka	Caucasian	51	34	16	1	68	38	26	4	0.872
Vieira et al.	2018	Brazil	Caucasian	119	53	49	17	112	61	41	10	0.419
Li et al.	2018	China	Asian	120	44	53	23	120	31	66	23	0.250
Sansoni et al.	2016	Italy	Caucasian	110	53	44	13	110	44	51	15	0.971
Colombini et al.	2015	Italy	Caucasian	267	117	120	30	254	101	117	36	0.821
Vieira et al.	2014	Brazil	Caucasian	121	54	50	17	131	75	46	10	0.434
Cervin et al.	2014	Mexico	Caucasian	100	20	65	15	100	32	51	17	0.664
Kelempisioti et al.	2011	Finland	Caucasian	150	81	57	12	246	111	119	16	0.032
Eser et al.	2010	Turkey	Caucasian	150	81	52	17	150	67	67	16	0.902
Eskola et al.	2010	Denmark	Caucasian	66	29	27	10	154	45	90	19	0.012
Nunes FTB et al.	2007	Brazil	Caucasian	66	9	54	3	88	61	27	0	0.089
Chen et al.	2007	China	Asian	81	18	51	12	101	36	48	17	0.883
Noponen-Hietala et al.	2003	Finland	Caucasian	29	11	12	6	56	25	26	5	0.630
TaqI												
Chen et al.	2012	China	Asian	81	79	2	0	101	86	14	1	0.617
Cheung et al.	2006	China	Asian	388	354	33	1	191	183	8	0	0.768
Oishi et al.	2003	Japan	Asian	39	31	8	0	21	16	5	0	0.536
Xu et al.	2014	China	Asian	78	75	3	0	156	153	3	0	0.903
Yuan et al.	2010	China	Asian	178	156	22	0	284	256	28	0	0.382
Kawaguchi et al.	2002	Japan	Asian	116	79	37	0	89	72	17	0	0.319
Eskola et al.	2010	Denmark	Caucasian	66	29	28	9	154	57	74	23	0.898
Eser et al.	2010	Turkey	Caucasian	150	65	67	18	150	67	67	16	0.902
Noponen-Hietala et al.	2003	Finland	Caucasian	29	12	11	6	56	26	19	11	0.044
Cervin et al.	2014	Mexico	Caucasian	100	69	27	4	100	62	35	3	0.461
Yang et al.	2019	China	Asian	454	32	227	195	485	63	246	176	0.110
Vieira et al.	2018	Brazil	Caucasian	119	50	42	27	112	52	46	14	0.448
Li et al.	2018	China	Asian	120	114	6	0	120	109	11	0	0.599
ApaI												
Chen et al.	2012	China	Asian	81	44	28	9	101	43	46	12	0.955
Kawaguchi et al.	2002	Japan	Asian	116	51	48	17	89	41	39	9	0.951
Yuan et al.	2010	China	Asian	178	58	100	20	284	128	129	27	0.500
Zawilla et al.	2014	Egypt	Caucasian	84	17	48	19	60	34	22	4	0.863
Yang et al.	2019	China	Asian	454	34	203	217	485	50	191	244	0.170
Vieira et al.	2018	Brazil	Caucasian	119	37	64	18	112	39	59	14	0.249
Li et al.	2018	China	Asian	120	13	47	60	120	16	48	56	0.273

### 3.2 Results of the overall meta-analysis

**Figure 2:**
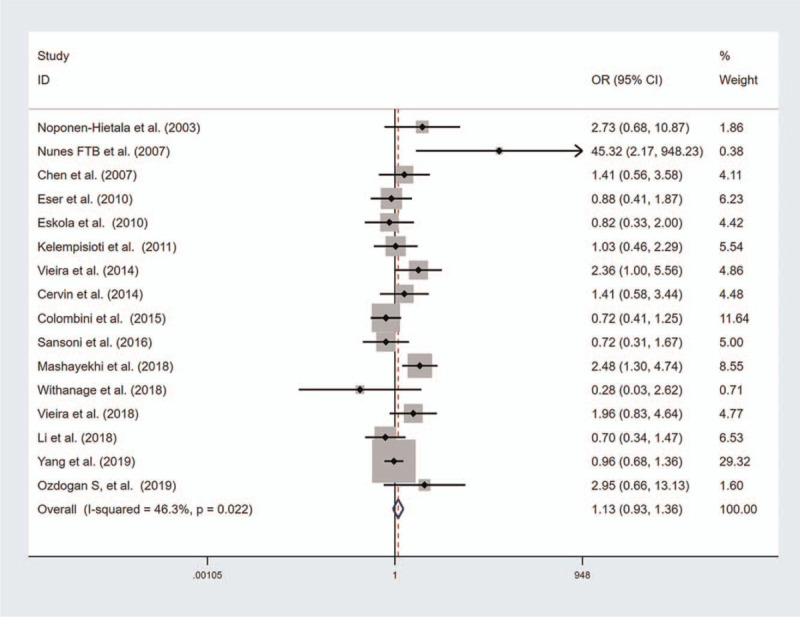
Forest plot of the association between vitamin D receptor (VDR) FokI polymorphism and disc degeneration risk (Homozygote model).

**Figure 3:**
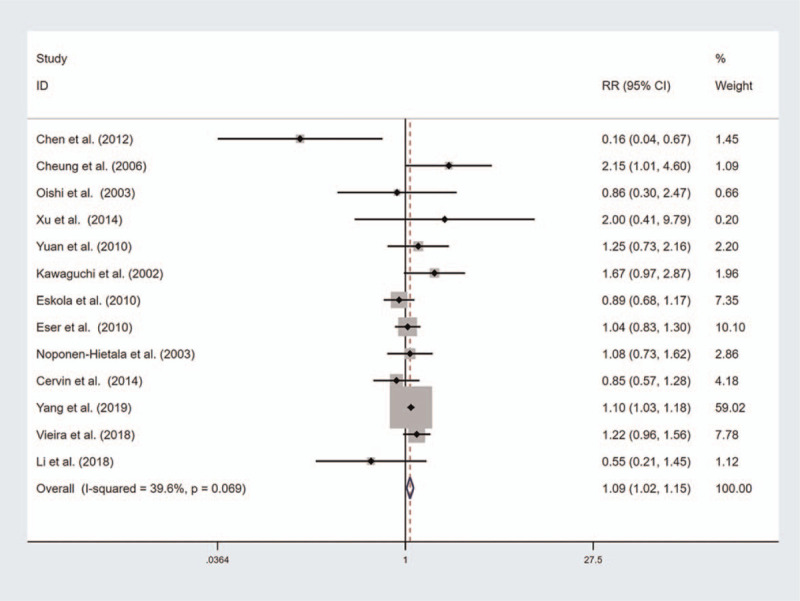
Forest plot of the association between vitamin D receptor (VDR) TaqI polymorphism and disc degeneration risk (Additive model).

**Figure 4:**
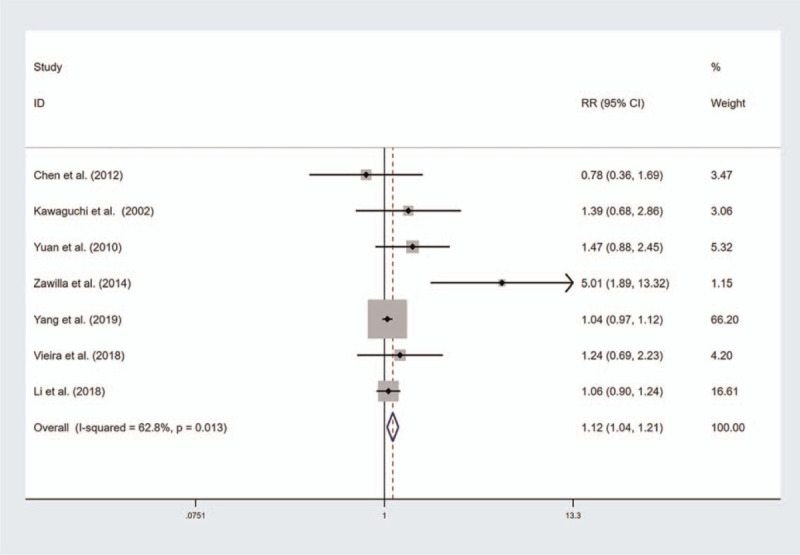
Forest plot of the association between vitamin D receptor (VDR) ApaI polymorphism and disc degeneration risk (Homozygote model).

A summary of the meta-analysis results for the association between VDR gene polymorphisms and risk of disc degeneration is shown in Table 2. No significant association was found between VDR FokI polymorphism and risk of disc degeneration (Fig. 2). However, the results of subgroup analyses by ethnicity showed a significant association of VDR FokI mutation with disc degeneration risk in Caucasians (Recessive model, OR with 95%CI 1.301, [1.041, 1.626], Additive model, OR with 95%CI 1.119, [1.006, 1.245]). There was a significant association between VDR TaqI mutation and risk of disc degeneration (Homozygote model, OR with 95%CI 1.167, [1.050, 1.290]; Recessive model, OR with 95%CI 1.194, [1.034, 1.378]; Additive model, OR with 95%CI, 1.085, [1.020, 1.154] Fig. 3). However, the results of subgroup analyses by ethnicity showed a significant association of VDR TaqI mutation with disc degeneration risk in Asians but not in Caucasians. There was a significant association between VDR ApaI mutation and risk of disc degeneration and subgroup analyses by ethnicity showed a significant association in Caucasians and in Asians (Fig. 4). The results of subgroup analyses by ethnicity are shown in Table 3.

**Table 2:** Summary of the meta-analysis results for the association between VDR gene polymorphisms and risk of disc degeneration.

Models	OR, 95% CI	Heterogeneity	*Z* and *P*
FokI			
Homozygote model	1.126, [0.932,1.360]	Heterogeneity chi-squared = 27.92 (d.f. = 15) p = 0.022, I-squared = 46.3%	z = 1.23, *P* = .218
Heterozygote model	1.100, [0.811, 1.491]	Heterogeneity chi-squared = 70.85 (d.f. = 15) p = 0.000, I-squared = 78.8%	z = .61, *P* = .540
Dominant model	1.159, [0.862, 1.559]	Heterogeneity chi-squared = 75.31 (d.f. = 15) p = 0.000 I-squared = 80.1%	z = .98, *P* = .328
Recessive model	1.148, [0.972, 1.355]	Heterogeneity chi-squared = 21.26 (d.f. = 15) p = 0.129 I-squared = 29.4%	*z* = 1.62, *P* = .104
Additive model	1.070, [0.981, 1.168]	Heterogeneity chi-squared = 64.31 (d.f. = 15) p = 0.000 I-squared = 76.7%	*z* = 1.52, *P* = .128
TaqI			
Homozygote model	1.167, [1.050, 1.296]	Heterogeneity chi-squared = 3.50 (d.f. = 12) p = 0.991 I-squared = 0.0%	*z* = 2.88, *P* = .004
Heterozygote model	1.051, [0.970, 1.137]	Heterogeneity chi-squared = 20.50 (d.f. = 12) p = 0.058 I-squared = 41.5%	*z* = 1.22, *P* = .224
Dominant model	1.051, [0.994, 1.112]	Heterogeneity chi-squared = 19.89 (d.f. = 12) p = 0.069 I-squared = 39.7%	*z* = 1.74, *P* = .082
Recessive model	1.194, [1.034, 1.378]	Heterogeneity chi-squared = 3.60 (d.f. = 12) p = 0.990 I-squared = 0.0%	*z* = 2.42, *P* = .015
Additive model	1.085, [1.020, 1.154]	Heterogeneity chi-squared = 19.88 (d.f. = 12) p = 0.069 I-squared = 39.6%	*z* = 2.59, *P* = .009
ApaI			
Homozygote model	1.122, [1.038, 1.213]	Heterogeneity chi-squared = 16.13 (d.f. = 6) p = 0.013 I-squared = 62.8%	*z* = 2.91, *P* = .004
Heterozygote model	1.113, [1.038, 1.192]	Heterogeneity chi-squared = 16.79 (d.f. = 6) p = 0.010 I-squared = 64.3%	*z* = 3.02, *P* = .003
Dominant model	1.076, [1.030, 1.124]	Heterogeneity chi-squared = 24.11 (d.f. = 6) p = 0.000 I-squared = 75.1%	*z* = 3.28, *P* = .001
Recessive model	1.040, [0.930, 1.163]	Heterogeneity chi-squared = 8.21 (d.f. = 6) p = 0.223 I-squared = 26.9%	*z* = 0.68, *P* = .494
Additive model	1.065, [1.012, 1.121]	Heterogeneity chi-squared = 23.28 (d.f. = 6) p = 0.001 I-squared = 74.2%	*z* = 2.44, *P* = .015

**Table 3:** The results of subgroup analyses by ethnicity.

Ethnicity	Homozygote model	Heterozygote model	Dominant model	Recessive model	Additive model
FokI					
Asian	0.952, [0.706, 1.283]^∗^	0.961, [0.744, 1.241]	0.960, [0.756, 1.220]	0.982, [0.766, 1.259]	0.978, [0.841, 1.138]
Caucasian	1.259, [0.987, 1.606]	1.023, [0.877, 1.194]	1.070, [0.924, 1.238]	1.301, [1.041, 1.626]	1.119, [1.006, 1.245]
Overall	1.126, [0.932, 1.360]	1.006, [0.882, 1.149]	1.039, [0.917, 1.177]	1.148, [0.972, 1.355]	1.070, [0.981, 1.168]
TaqI					
Asian	1.159, [1.053, 1.276]	1.123, [1.023, 1.232]	1.087, [1.024, 1.155]	1.175, [1.004, 1.375]	1.111, [1.037, 1.191]
Caucasian	1.190, [0.872, 1.625]	0.941, [0.814, 1.087]	0.983, [0.874, 1.106]	1.255, [0.902, 1.746]	1.030, [0.908, 1.169]
Overall	1.167, [1.050, 1.290]	1.051, [0.970, 1.137]	1.051, [0.994, 1.112]	1.194, [1.034, 1.378]	1.085, [1.020, 1.154]
ApaI					
Asian	1.070, [0.993, 1.154]	1.078, [1.001, 1.161]	1.047, [1.002, 1.094]	1.000, [0.892, 1.120]	1.032, [0.980, 1.088]
Caucasian	2.048, [1.260, 3.331]	1.287, [1.067, 1.551]	1.282, [1.095, 1.502]	1.744, [1.018, 2.986]	1.353, [1.124, 1.629]
Overall	1.122, [1.038, 1.213]	1.113, [1.038, 1.192]	1.076, [1.030, 1.124]	1.040, [0.930, 1.163]	1.065, [1.012, 1.121]

### 3.3 Test for heterogeneity

There was a significant heterogeneity between VDR FokI polymorphism and risk of disc degeneration except in Recessive model: Heterogeneity chi-squared = 21.26 (d.f. = 15) *P* = .129, I-squared = 29.4%. No significant heterogeneity between VDR TaqI polymorphism and risk of disc degeneration was found in all models. There was a significant heterogeneity between VDR FokI polymorphism and risk of disc degeneration except in Recessive model: Heterogeneity chi-squared = 8.21 (d.f. = 6) *P* = .223, I-squared = 26.9%. We assessed the source of heterogeneity by region, publication year, ethnicity, and sample size. However, we did not observe any sources that contributed to the substantial heterogeneity.

### 3.4 Sensitivity analysis

We conducted sensitivity analyses to ascertain the primary origin of the heterogeneity. Through sensitivity analysis, the present study showed that no individual studies were found to significantly influence the pooled effects in each genetic model.

### 3.5 Publication bias

**Figure 5:**
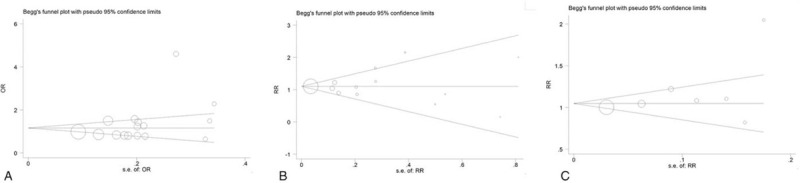
Begg funnel plot for assessing potential influence of publication bias on the observed association between the vitamin D receptor (VDR) polymorphisms and disc degeneration risk (Additive model, A, FokI; B TaqI; C, ApaI).

Funnel plot was generated to assess publication bias (Fig. 5). Begg test and Egger's test were performed to evaluate funnel plot symmetry statistically. The results showed no publication bias: Begg test *P* = .079 and Egger test *P* = .201 for VDR FokI; Begg test *P* = .855 and Egger test *P* = .739 for VDR TaqI; Begg test *P* = .230 and Egger test *P* = .207 for VDR ApaI.

## 4 Discussion

The disc degeneration has been proved to be a multifactorial result, influenced by environmental and genetic determinants. A number of environmental factors such as age, obesity, excessive mechanical loading, injury, vibration, and smoking status, were reported to have an impact on disc degeneration.^[38]^ However, more and more evidence showed that genetic factors may play a critical role in occurrence of disc degeneration.^[39]^ Among these genetic factors, the allelic variants of the gene encoding VDR, include TaqI (rs731236), FokI (rs2228570) and ApaI (rs7975232) have been reported to be associated with disc degeneration risk. Videman et al. performed a population-based Finnish Twin cohort study and firstly reported that specific VDR alleles were associated with intervertebral disc degeneration.^[40]^

After that a series of studies with limited sample sizes have explored the association between VDR gene polymorphisms and disc degeneration risk, but the results still remain controversial. Several studies^[23,25,28]^ have proved the association between VDR gene polymorphisms and disc degeneration risk but other studies^[31–33]^ failed to find such associations.

Several meta- analyses have been performed but results still remain extremely controversial. Xu et al^[6]^ conducted a meta-analysis based on a total of 9 studies for TaqI, 5 studies for FokI, and 3 studies for ApaI and they reported that VDR (TaqI, FokI, ApaI) gene polymorphisms were not significantly associated with the risk of disc degeneration. Jiang et al^[9]^ erformed a meta-analysis based on 14 studies and concluded that TaqI, FokI, and ApaI polymorphisms of VDR gene were not significantly associated with disc degeneration susceptibility. Nong et al^[10]^ performed a meta-analysis based on all papers published until December 2014 and found no obvious association between VDR FokI and ApaI polymorphisms and disc degeneration susceptibility. A recent review analyzed seven meta-analyses and concluded that there is no evidence of an association between FokI polymorphism and IDD in the general population.^[12]^ However, such a conclusion is not supported other meta-analyses: a meta-analysis performed by Chen et al^[8]^ demonstrated that the VDR FokI polymorphism may be associated with disc degeneration susceptibility among Caucasians; Pabalan et al^[11]^ performed a meta-analysis and found that VDR ApaI polymorphism may be a protective role in disc degeneration but the VDR FokI polymorphism may be ethnic and gender specific.

Considering a large number of novel case-control studies have been published and the limitations of previous studies, we conducted this meta-analysis in a comprehensive way to drive a more precise estimation of association between VDR TaqI, FokI, and ApaI polymorphisms and disc degeneration risk. Finally a total of 23 studies published from 2003 to 2019 were included in this meta-analysis, to the best of our knowledge this is the most comprehensive meta-analysis at present. Based on the available evidence at present this meta-analysis found VDR FokI polymorphisms was associated with disc degeneration risk among Caucasians but not Asians, VDR TaqI polymorphisms was associated with disc degeneration risk among Asians but not Caucasians, there was also an obvious association between VDR ApaI polymorphism and disc degeneration risk among Asians and Caucasians. Significant heterogeneity was detected in our study for FokI and ApaI analysis and we assessed the source of heterogeneity by region, publication year, and sample size. However, we did not observe any sources that contributed to the substantial heterogeneity. The sensitivity analyses and publication bias results confirmed the reliability of these conclusions.

Several potential limitations of this meta-analysis should be discussed:

(1) selection bias may have occurred because only studies in English or Chinese were selected;
(2) there was a significant heterogeneity;
(3) the specific mechanism underlying the relationship between VDR gene polymorphism and disc degeneration risk is still not entirely clear.

Despite the limitations listed above, this study has some clear advantages:

(1) this is most comprehensive meta-analysis based on 23studies at present;
(2) sub-group analysis stratified by ethnicity was performed;
(3) sensitivity analysis was performed;
(4) no publication bias was detected;
(5) the well-designed search and selection method significantly increased the statistical power of this meta-analysis.

## 5 Conclusion

In summary, based on the most updated information, we drew a more reliable conclusion on the influence of VDR gene polymorphisms on disc degeneration. The results of our meta-analysis indicate that VDR FokI polymorphisms was associated with disc degeneration risk among Caucasians but not Asians, VDR TaqI polymorphisms was associated with disc degeneration risk among Asians but not Caucasians, while VDR ApaI polymorphism was associated with disc degeneration risk among Asians and Caucasians. (SDC: individual data: .).

## Acknowledgments

We are grateful to all authors.

## Author contributions

**Conceptualization:** jing xue.

**Data curation:** jing xue.

**Formal analysis:** Quan Gong.

**Methodology:** yueming song.

**Project administration:** Limin Liu.

**Software:** Hao Liu.

**Writing – original draft:** jing xue.

**Writing – review & editing:** yueming song, Tao Li.

## Supplementary Material

Supplemental Digital Content
