# Supplementary material for: Vitamin D receptor gene polymorphisms and risk of intervertebral disc degeneration: An updated meta-analysis based on 23 studies
Source: Medicine (Baltimore). 2021 May 21;100(20):e25922. doi: 10.1097/MD.0000000000025922 (PMC8136998; doi:10.1097/MD.0000000000025922)
Supplement: Supplemental Digital Content [file medi-100-e25922-s001.doc]

The individual data of all included studies.

| **Study** | **Year** | **Region** | **Ethnicity** | **Sex** | **Age（years）** | **Study design** | **Number**（**cases/controls**） | **Disease** | **Diagnostic criteria** | **Genotyping metod** |
| --- | --- | --- | --- | --- | --- | --- | --- | --- | --- | --- |
| Yang et al.[17] | 2019 | China | Asian | Both | case:51.6  control:54.2 | PBS | 454/485 |  | Clinical examinations，MRI | SNaPshot |
| Ozdogan S, et al. [19] | 2019 | Turkey | Caucasian | Both | case:37.33±8.26  control:34.81±4.53 | HBS | 45/49 | LDD | MRI | PCR |
| Mashayekhi et al.[15] | 2018 | Iran | Caucasian | Both | case:37.32±8.81  control:35.9±7.63 | HBS | 180/230 | LDD | MRI | PCR-PFLP |
| Withanage et al.[16] | 2018 | Sri Lanka | Caucasian | Both | case: 41.3±14.6  control: 43.3±15.4 | PBS | 51/68 | LBP,LDD | MRI | PCR-PFLP |
| Vieira et al.[18] | 2018 | Brazil | Caucasian | Both | case: 40  control: 32 | HBS | 119/112 | DD | MRI | PCR-PFLP |
| Li et al.[21] | 2018 | China | Asian | Both | case:54.29±15.46  control:55.21±15.83 | PBS | 120/120 | LDD | MRI | SNaPshot |
| Sansoni et al.[20] | 2016 | Italy | Caucasian | both | case: 42.1 ± 9.3  control: 41.4 ± 7.9 | HBS | 110/110 | LDD | MRI | NM |
| Colombini et al.[28] | 2015 | Italy | Caucasian | Both | NM | HBS | 267/254 | LDD | MRI | PCR-PFLP |
| Vieira et al.[25] | 2014 | Brazil | Caucasian | Both | case:  male:46.0±5.4  female:45.2±5.9  control:  male:33.8±8.2  female:33.9±8.1 | HBS | 121/131 | LDD | MRI | PCR-PFLP |
| Cervin et al.[24] | 2014 | Mexico | Caucasian | Both | case:39.22±6.88  control：39.13±6.80 | HBS | 100/100 | LDD | MRI | PCR-PFLP |
| Xu et al. [27] | 2014 | China | Asian | Both | case:42.5±12.7  control：39.4±13.6 | HBS | 78/79 | LDD | MRI | PCR-PFLP |
| Zawilla et al. [26] | 2014 | Egypt | Caucasian | Both | case:44.2±11.28  control:43.3±10.57 | HBS | 84/60 | LDD | MRI | PCR-PFLP |
| Chen et al.[29] | 2012 | China | Asian | Both | case:42.7  control:38.4 | PBS | 81/101 | LDD | MRI | PCR-PFLP |
| Kelempisioti et al.[32] | 2011 | Finland | Caucasian | Both | 40.3 | HBS | 150/246 | LDD | MRI | SNaPshot |
| Eser et al.[30] | 2010 | Turkey | Caucasian | NM | 20-30 | HBS | 150/150 | LBP | MRI | PCR-PFLP |
| Eskola et al. [31] | 2010 | Denmark | Caucasian | Both | 13.1±0.4 | PBS | 66/154 | LDD | MRI | SNaPshot |
| Yuan et al.[36] | 2010 | China | Asian | Both | case:48.5±13.1  control:40.6±15.8 | HBS | 178/284 | LDD | CT | PCR-PFLP |
| Nunes FTB et al.[34] | 2007 | Brazil | Caucasian | Both | case:38,control:41 | HBS | 66/88 | LDD | MRI | PCR |
| Chen et al.[37] | 2007 | China | Asian | Both | case:42.7,  control:38.4 | PBS | 81/101 | LDD | MRI | PCR-PFLP |
| Cheung et al.[23] | 2006 | China | Asian | Both | 18-55 | PBS | 388/191 | LDD | MRI | PCR |
| Noponen-Hietala et al.[33] | 2003 | Finland | Caucasian | Both | case:59,  control:43 | PBS | 29/56 | LSS | MRI,CT | PCR |
| Oishi et al. [35] | 2003 | Japan | Asian | Female | 73.2±5.9 | HBS | 39/21 | LBP | MRI | PCR |
| Kawaguchi et al.[22] | 2002 | Japan | Asian | Both | 22 | HBS | 116/89 | LBP | MRI | PCR-RFLP |

HBS: hospital-based study PBS: population-based study NM: not mentioned PCR:polymerase chain reaction PCR-PFLP: polymerase chain reaction-restriction fragment length polymorphism LDD: lumbar disc degeneration LBP: low back pain LSS: lumbar spinal stenosis DD: disc degeneration
